# Supplementary material for: MicroRNA-26b Attenuates Platelet Adhesion and Aggregation in Mice
Source: Biomedicines. 2022 Apr 23;10(5):983. doi: 10.3390/biomedicines10050983 (PMC9138361; doi:10.3390/biomedicines10050983)
Supplement: Supplementary file 1 [file biomedicines-10-00983-s001.zip › biomedicines-1670250-supplementary.pdf]

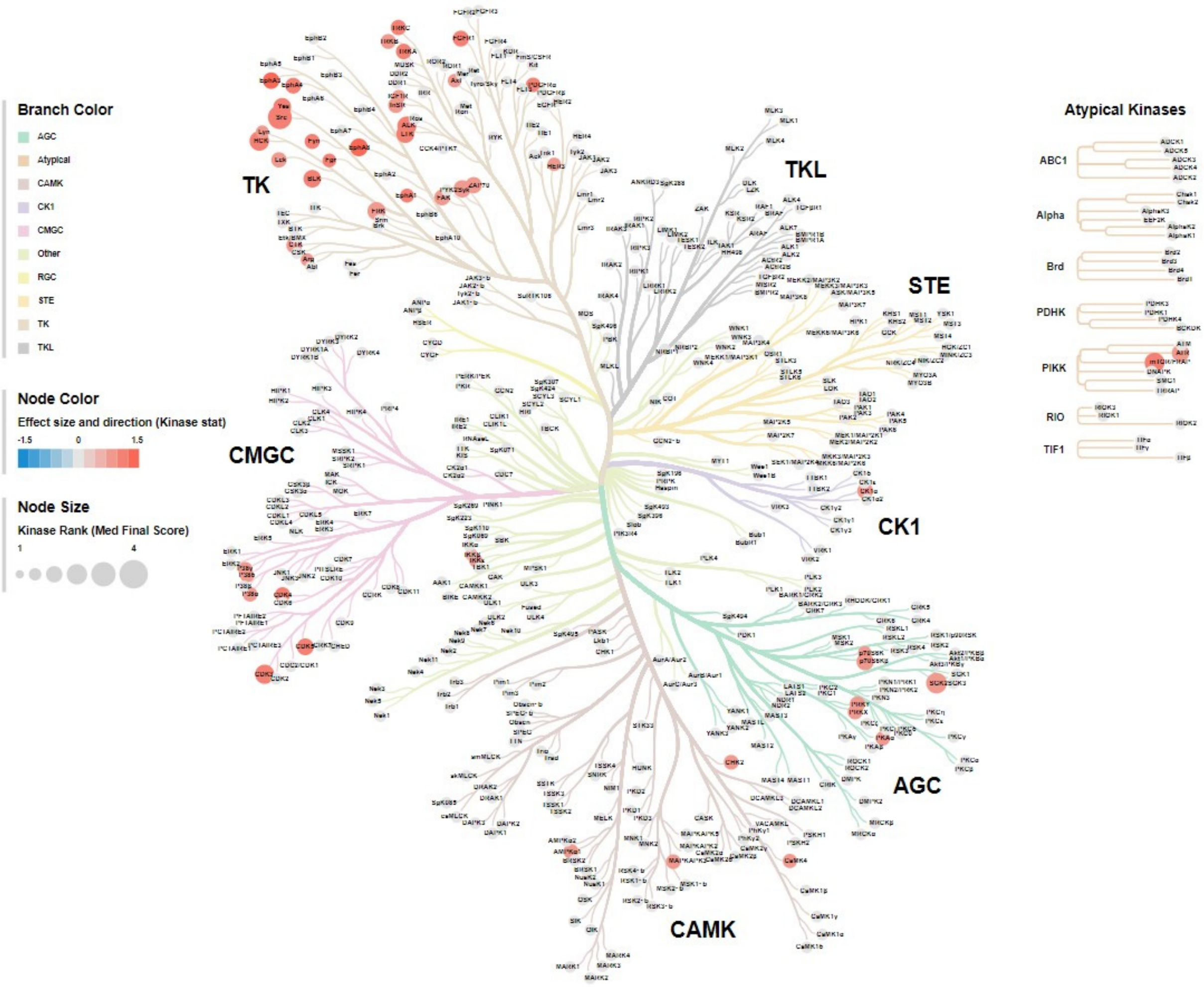

Supplementary Figure S1. CORAL kinome tree for activated platelets isolated from miR-26b knockout mice compared to wildtype control. CORAL kinome tree demonstrating effect size and direction (*Apoe*$^{-/-}$*Mir26b*$^{-/-}$ vs. *Apoe*$^{-/-}$) and node size from PTK and STK (*n* = 3) PamChip arrays.
